# How Do Nocebo Phenomena Provide a Theoretical Framework for the COVID-19 Pandemic?

**DOI:** 10.3389/fpsyg.2020.589884

**Published:** 2020-10-30

**Authors:** Martina Amanzio, Jeremy Howick, Massimo Bartoli, Giuseppina Elena Cipriani, Jian Kong

**Affiliations:** ^1^Department of Psychology, University of Turin, Turin, Italy; ^2^European Innovation Partnership on Active and Healthy Ageing (EIP-AHA), Brussels, Belgium; ^3^Faculty of Philosophy, University of Oxford, Oxford, United Kingdom; ^4^Department of Psychiatry, Massachusetts General Hospital, Harvard Medical School, Charlestown, MA, United States

**Keywords:** COVID-19 pandemic, negative expectation, nocebo effects in randomised controlled trials, nocebo responses in brain imaging studies, mood changes, psychosocial context

## Abstract

The COVID-19 pandemic is a major health issue, which leads to psychological and behavioural changes. In particular, among various negative feelings, fear seems to be one of the main emotional reactions that can be as contagious as the virus itself. The actual pandemic is likely to function as an important stressor, especially in terms of chronic anxiety and lack of control over the succession of unforeseeable environmental events. In this direction, the psychological impact of previous quarantine measures showed important negative psychological effects, including post-traumatic stress symptoms (PTTS) with long-lasting effects. The presence of psychological discomfort and disturbances due to negative contextual factors can be studied using the nocebo phenomenon as a possible theoretical explanatory framework. Although in the absence of studies linking nocebo to Covid-19 and data-driven evidence, the context of the actual pandemic may be seen as a fertile ground for amplified discomfort and anxiety. The media provide dramatic and negative descriptions and often present conflicting sources of information, which can lead to physical and mental health problems, diminishing response to treatment. This can be worse when supported by conspiracy theories or misinformation. The aim of this perspective review is to propose a new theoretical framework for the COVID-19 pandemic, which should be supported by future empirical studies. In particular, the negative contextual factors, which can predispose individuals to psychological distress and the onset of the nocebo phenomena will be presented here, in order to suggest possible guidelines to mitigate the devastating effects of COVID-19.

## IntroductIon

The COVID-19 pandemic includes a perfect storm in which powerful nocebo effects may be flourishing. The nocebo effect can be mediated by situational-contextual factors (such as verbal information and suggestions, healthcare beliefs and health professional interactions, exposure to negative media campaigns, or previous personal experience) and by individual factors.

In Hahn and Kleinman (1983) published, in the prestigious Medical Anthropology Quarterly, a short article on the effects of belief. In particular, the authors underlined that beliefs may “kill” and beliefs may “heal” and what a person believes within a society plays a significant role both in producing disease and as a remedy. The authors illustrated different forms of the nocebo effect, as beliefs influence outcomes, particularly in the absence of specific events or communications: fear of heart disease increases the risk of ischemic attack; similarly, depressive states – i.e., a generalised sense of impotence – increase the probability of death as a result of ischemic events. Moreover, it is important to note how Cannon (1942) had already previously defined the phenomenon of “voodoo death” as a dramatic nocebo effect, following the induction of a pervasive state of terror. Prolonged stress events due to different adverse environmental contexts can cause the collapse of the neurovegetative balance and this can be so serious that it paralyses vital functions and induces death, even in the absence of organic lesions. In particular, the death may be caused by lasting and intense action of the sympathico-adrenal system. Since Cannon’s observations, accumulated evidence supported his concept of “voodoo death” and nowadays it is considered as a real phenomenon, but far from being limited to ancient peoples. It can be defined as a basic biological principle that provides an important clue to understand the phenomenon of sudden death, as well as to provide an explanation for neurovisceral diseases (Samuels, 2007).

Subsequently, the research data and experiments on the nocebo effect have multiplied, substantially confirming the hypotheses of the previous authors, demonstrating important novel relationship between stress and emotion in the field of the neurobiology of pain (Amanzio et al., 2016a). Nocebo phenomena have a detrimental effect on health in terms of psychosomatic factors produced mainly by psychosocial aspects, such as negative treatment expectations or prognosis. Recently, nocebo has become a popular research topic, as it compromises treatment outcome and reduces adherence to therapy (Howick et al., 2018). In addition, negative expectations can increase stress and anxiety levels, which can affect our health and well-being (Kong and Benedetti, 2014).

Although in the absence of studies linking the nocebo effect to COVID-19 and data-driven evidence, the outbreak of the actual pandemic, and other past epidemics, may be a perfect scenario for an amplified nocebo effect to occur. In particular, when individuals feel the lack of control of a new situation and the perceived high level of contagion risk, the lack of information to refer to, the lack of available treatments or vaccines, and the spread of negative news. In addition, quarantine measures caused post-traumatic stress symptoms (PTSS), confusion, anxiety, and anger associated with acute stress reactions and post-traumatic stress disorder (Brooks et al., 2020).

The COVID-19 pandemic led to negative emotions, such as fear and anxiety (Liu et al., 2020). In particular, recently, intense anxiety and PTSS have been described among the Chinese population, especially Wuhan residents, due to the number of infections increasing, the lack of clear and definite information of virus from the media, the shortage of medical workers and resources, and the lack of masks and protecting supplies in the marketplace (Kang et al., 2020). In addition, the social distancing and isolation that accompany long-term lockdowns might be a risk factor for anxiety, addictive, and mood disorders (Sani et al., 2020).

In the current pandemic, important stressors are mainly due to uncertainty and changes in the environment and, in some cases, lack of activity to shift attention away from negative news and information, which trigger negative thoughts and expectations. In this direction, contextual factors, such as social networks and media, flood people with dramatic and mostly negative information. They present conflicting and confusing sources of information, often supported by conspiracy theories and misinformation. These news sources represent a possible breeding ground for psychological distress and a great burden for individuals. It is important to note that conflicting information are associated with increased stress. Misplaced expectations (probably one type of conflicting information) can lead to anxiety and/or depression if and when authorities apply the COVID-19 lockdown more rigorously (Torales et al., 2020). In particular, stress associated with negative expectations, which can be a fertile substrate for the onset of a nocebo effect, can produce significant physiological changes in the human body, including sleep disorders, respiratory complications, circulatory stress, digestive disorders, muscle tension, and pain (Liu et al., 2020). These symptoms are likely to further aggravate the prognosis of individuals with COVID-19.

Given the hypothesised importance of the negative contextual factors, which can predispose individuals to psychological distress and the onset of the nocebo phenomena, studies characterising nocebo phenomena in clinical trials and in brain imaging experiments will be presented in order to provide an interesting theoretical framework in the current COVID-19 pandemic. Finally, possible guidelines to mitigate the devastating effects of COVID-19 will be suggested.

## Negative Contextual Factors Predisposing Individuals To Psychological Distress

Previous coronavirus epidemics caused an increase in stress levels and neuropsychiatric implications, − i.e., mental disorders that are the sequelae of brain damage or disease, in patients admitted to hospital for Severe acute respiratory syndrome due to coronavirus (SARS-CoV) or Middle East respiratory syndrome (MERS-CoV; Rogers et al., 2020) – as also reported by WHO (2020) for the actual pandemic. In line with those above reported, past epidemics had been related to several and long-lasting psychiatric consequences (Kępińska et al., 2020).

Feelings of growing concern had also been aggravated by conflicting opinions among experts on pandemics. For most countries, an underestimation of the COVID-19 phenomenon had been observed, together with the presence of conflicting information (such as on the epidemic-pandemic). Using Italy as an example, some virologists underestimated COVID-19, describing it as a “trivial influence.” Meanwhile, other experts strongly contrasted this information by warning the population of the contagion risk and gravity of SARS-CoV-2. In addition, during the initial phase after the lockdown (phase 2), some experts reported a reduction in COVID-19 virulence, which was not supported by scientific or clinical evidence.

In the United Kingdom and United States, initially, governments avoided placing restrictive measures, such as lockdown on the population, claiming that “*herd immunity*” would have been the most natural outcome. At the same time, important virologists from the Imperial College London and WHO discouraged this path providing precise instructions on how to contain the spread of COVID-19 through the same restrictive measures previously taken by China and Italy.

Furthermore, negative distressing information presented during phase 1 of the pandemic (see Table 1 for a list of examples), mainly consisted of: (1) media repeating information on the number of infections and exitus, (2) the absence of protective aids to fight infection for the public and medical-healthcare personnel alike, and the absence of vital biomedical devices to fight SARS-CoV-2, (3) total lack of scientific evidence on the new viral agent and consequent absence of diagnostic and prognostic perspectives, (4) stories of patients deprived of any contact with loved ones, especially at the time of aggravation of the symptoms that led to death, and (5) the repeated presentation of images of patients under anaesthesia and in intensive care units, and coffins carried on military trucks.

**Table 1:** Examples of negative information.

Information	Publication date	Source
The pandemic alarm will last a long time	May 14th, 2020	WHO
Covid-19: after the lockdown in Korea, China and Germany the contagions increase	May 12th, 2020	WHO
Over 4 million infections in the world. Three out of four in EU countries and the US	May 11th, 2020	John Hopkins University
People living longer and healthier lives but COVID-19 threatens to throw progress off track	May 13th, 2020	WHO
Preparing for a long, hot summer with COVID-19	May 11th, 2020	WHO, EU Region
US$675 million needed for new coronavirus preparedness and response global plan	February 5th, 2020	WHO
WHO announces COVID-19 outbreak a pandemic	March 12th, 2020	WHO

Moreover, misinformation increased confusion and uncertainty. For example, the events around the world associated with the COVID-19 pandemic fuelled strong states of anxiety making people more willing to believe in conspiracies (Grzesiak-Feldman, 2013). Indeed, Swami et al. (2016) reported that stressed individuals are more likely than others to believe in conspiracy theories. Moreover, investigators found that promoting anxiety in people also makes them more conspiracy-minded (Jolley and Douglas, 2017; Jolley et al., 2020). When personal alienation or anxiety are combined with the feeling of a dangerous society, people are more likely to believe in conspiracy theories, thus increasing their sense of powerlessness and making them feel even worse.

## Theoretical Framework of Nocebo Effects and Responses. Possible Association Between Psychological Distress and the onset of the Nocebo Phenomena

Studies and results characterising nocebo phenomena in experimental settings, clinical trials, and in brain imaging experiments can provide an interesting theoretical framework in the current COVID-19 pandemic. The neuroscience of pain, stress, and emotion underlined that the hyperalgesic nocebo effect appears to be attributable to complex biochemical and neuroendocrine mechanisms that link anxiety to pain involving the activation of the cholecystokininergic system. In particular, previous studies suggested that anxiety produced by negative expectancy may play a key role in the nocebo effect. In particular, using nocebo hyperalgesia as an example, negative verbal suggestions – about an impending pain increase – induce anticipatory anxiety and an hyperactivity of the hypothalamic-pituitary-adrenal axis (HPA), leading to the activation of cholecystokinin (CCK), anti-opioid peptide, which, in turn, facilitate pain transmission (Benedetti et al., 2006, 2007).

Furthermore, considering how HPA hyperactivity and nocebo hyperalgesia can be antagonised by benzodiazepine diazepam, Benedetti et al. (2006) suggested how anxiety could be involved in these effects.

In this direction, individuals with pathologies such as anxiety and depression, and those with a tendency towards somatization, had been found to be more likely to develop nocebo effects and responses (Wells and Kaptchuk, 2012). In particular, anxiety, depression, and somatization are considered some of the psychological factors involved in nocebo related side effects in Randomized Clinical Trials (RCTs; Barsky et al., 2002). As reported by clinicians, anxiety can lead to side effects as its somatic symptoms, such as tachycardia, dyspnea, and sweating (Ferguson, 1993).

Neuroimaging data showed how the affective-cognitive pain circuit was involved, with different modulation, in both the nocebo hyperalgesia and the placebo analgesia (Amanzio et al., 2013; Palermo et al., 2015).

In a functional magnetic resonance imaging study, Kong et al. (2008) analysed the brain regions involved in the nocebo response following an expectation of hyperalgesia. Their results showed an activation of many areas, such as bilateral dorsal anterior cingulate cortex, orbital prefrontal cortex, superior parietal lobe, hippocampus, insula, right claustrum/putamen, left frontal and parietal operculum, middle and superior temporal gyrus, lateral prefrontal gyrus, and medial frontal gyrus.

Neuroimaging data related to pain anticipation highlighted how negative expectancies had a substantial effect on cortical mechanisms.

In particular, a cognitive, affective, and motivational neural reaction, essential for survival, can be activated by negative expectations and psychosocial stimuli. Moreover, negative anticipation modulatory neural activations, implicated in salience detection, emotion/arousal, autonomic responses, and executive functioning, may underlie increased levels of mood-changes related to fear, anxiety, and hypervigilance (Palermo et al., 2015).

Randomized Clinical Trials are useful in studying the role of a patient’s psychosocial environment and the context in which therapies are administered on subsequent negative outcomes. The evaluation of adverse events (AEs) in the placebo group, matched with a specific psychotropic drug, provides an important perspective for understanding this phenomenon (Amanzio, 2015). Psychiatric patients, above all with mood and psychotic symptoms, represent an interesting population in order to study the nocebo effect. Indeed, AEs affect adherence and dropout rates among patients with psychiatric disorders in RCTs (Wahlbeck et al., 2001). Thus, AEs can be useful for an accurate description of patients with psychiatric diseases, who expect more negative clinical outcomes.

Moreover, the level of psychopathology, such as the severity of positive symptoms and signs of anxiety and depression, widely affected their perceptions and attribution of bodily sensations to medications (Hwang et al., 2010). Indeed, a higher level of psychiatric symptomatology makes patients more prone to express AEs manifested as nocebo-like effects (Palermo et al., 2019; Amanzio and Palermo, 2020). In addition, studying patients with pain conditions and neurodegenerative diseases would also be important, considering their clinical implications. In fact, as reported by a systematic review on nocebo effects in clinical trials by Amanzio et al. (2016b), neurological patients have a high probability of a negative outcome.

The reported findings may help to better understand the COVID-19-related distress due to excessive feelings and negative outcomes. In particular, understanding nocebo responses is important because they are substantial across disorders and may be associated with objective pathology and survival. Moreover, research on nocebo responses provides a way to investigate how the brain systems implicated in the processing of contextual information (such as threats) influence psychophysiology and clinically relevant outcomes, such as in the case of COVID-19. In addition, understanding how negative context and anticipatory negative expectancies influence outcomes in placebo groups of RCTs, in terms of AEs and dropout, will be essential to understand how people are now experiencing COVID-19-related symptomatology.

The negative information and harbingers of distress can be associated with the neuro-psychophysiological correlates observed in the nocebo effect and response through its cerebral underpinnings (the flipside of a positive outcome due to a placebo). Nocebo responses are associated with activity changes in brain areas, such as the amygdala, that are also involved in mood regulation (Freeman et al., 2015), and thus may worsen the stress/anxiety response to COVID-19.

In the presence of negative suggestions and nocebo effects associated with the SARS-CoV2 infection, the outcome of the disease can become more unfavourable, as reported for other diseases (Barsky, 2017). These more negative prognoses should be taken into greater consideration, especially in the elderly, with physical frailty and possible cognitive impairments, because they are at greater risk of SARS-CoV-2 infection and poorer prognosis.

The social distancing measures introduced to control the spread of COVID-19, while arguably required, also may exacerbates nocebo effects. A large body of evidence summarised by Howick et al. (2019) establishes that social isolation reduces mental health and increases mortality.

## Possible Ways To Decrease Negative Expectations, Stress and Anxiety Related To Covid-19

It is crucial to understand and minimise psychological distress during and after the pandemic by reducing negative expectations and anxiety about the risk of contagion. To do that, individuals should be informed on how interpret and manage situational and individual factors predisposing them to develop negative effects and symptoms to a greater extent. Moreover, encouraging a healthy lifestyle in order to strengthen the immune system and combat psychological and physical distress should be suggested.

In particular, regarding the individual factors that can predispose individuals to psychological distress, and the onset of the nocebo phenomena, two aspects should be highlighted: (1) the importance of maintaining the functional aspects of anxiety, as healthy and natural response to stressful circumstances, as useful to comply with the rules of conduct to reduce the risk of SARS-COV2 infection and (2) in contrast, high levels of anxiety about the risk of contagion, which can lead to a stress reaction causing PTTS, should be contrasted, for instance, by avoiding update on alarming news.

With regard to situational-contextual factors, in which possible nocebo effects may be flourishing, it should be highlighted how: (1) it may be helpful for individuals to translate negative messages and communication flows into neutral or positive information, (2) also focusing on positive information in order to decrease negative expectations, stress, and anxiety related to COVID-19 should be emphasised, (3) positive expectations should be supported by how new treatments and vaccine developments are making progress (see Table 2 as an example), and (4) the creation of a better balance of negative and positive information, focusing more on prevention, diagnostic, and prognostic perspectives (Vaughan and Tinker, 2009) should be encouraged. The authorised (evidence-based) information source will significantly reduce the spread and influence of fake or conflicting news (Tumpey et al., 2018).

**Table 2:** Examples of information possibly decreasing stress and anxiety.

Information	Publication date	Source
“The increase in the number of deaths continues to decline”	May 12th, 2020	Health Ministry, IT
“From large retailers 19 million surgical masks to citizens”	May 12th, 2020	Health Ministry, IT
“The people currently cured are 106,587”	May 11th, 2020	Health Ministry, IT
Brusaferro (NIH): “The contagion curve continues to decrease”	May 8th, 2020	National Institute of Health (Health Ministry, IT)
“40 years ago we defeated smallpox, now together we can also beat coronavirus”	May 8th, 2020	WHO
“The decline in ICU patients continues”	May 14th, 2020	Civil Protection Department, IT
“Discharged and healed, they exceed 50% of the total cases”	May 13th, 2020	Civil Protection Department, IT
Covid-19 Health Minister to G7 and EU: “Proceeding together on research, production and distribution of the vaccine”	May 7^th^, 2020	Communication of Health Minister, IT
Covid-19 “Great attention on re-openings. Listening to scientists and constant monitoring that is being done”	May 8th, 2020	Communication of Health Minister, IT
“Contracts to be entered into by the civil protection department for the supply of personal protection devices, medical and aid devices”	March 17th, 2020	Decree n. 18, March 17th, 2020
“Countries working to sustain population immunity to vaccine-preventable diseases during COVID-19 pandemic”	April 27th, 2020	WHO, European Region
PAHO Director: “Calls to accelerate and expand testing for COVID-19 in the Americas”	April 21st, 2020	Pan American Health Organization

ICU, intensive care unit.

Minimising nocebo effects might be an ethical requirement (Howick, 2020). Sharing of multiple sources of information, such as those regarding the COVID-19 pandemic, is necessary to stop the spread of disease. However, even if availability of information is the real defence against conflicting sources of information, they are, to some degree, unavoidable. In fact, the extent of this kind of information cannot be fully known yet, due to the uncertainty surrounding COVID-19. Some conflicting sources of information are created to spread anger and confusion, and some arise from haste and error. The former represent the most insidious form of COVID-19 information.

## Conclusions and Limitations of the Review

The most important limitation of this perspective review is the lack of empirical data on the association between the nocebo effect and COVID-19. However, the framework provided here may be an explorative and useful perspective to describe a phenomenon that is still new and unexplored nowadays.

During COVID-19, a possible nocebo response may be induced on a large scale due to negative information received from the media. These effects can be amplified by the environment, in particular by social isolation. Understanding how the nocebo effect can occur and minimise is a significant challenge, and may also be required ethically.

To do this, we should balance negative news with optimistic information, including how to prevent COVID-19, progresses in treatment, vaccines, and prevention, so that the vast majority of infected individuals will experience only minor symptoms. Although the COVID-19 era is an unavoidable breeding ground for the possible nocebo effect, stress management, exercise, and social contact – even remotely – can be promoted to mitigate them.

## Author Contributions

MA conceived of the content of this perspective review, wrote the first draft of the manuscript, and structured tables. JH and JK discussed the content of the review and proposed several additions to the text. MB and GEC wrote and edited the manuscript and revised tables. All authors contributed to the article and approved the submitted version.

### Conflict of Interest

The authors declare that the research was conducted in the absence of any commercial or financial relationships that could be construed as a potential conflict of interest.
